# A Treatise on Tenotomy as Applied to Club-Foot

**Published:** 1846-01

**Authors:** 


### ART. XIV.

*De Tenotomia Talipedibus applicata. Commentatio quam scripsit* CHR. WEIS.—*Havniæ*, 1844.

*A Treatise on Tenotomy as applied to Club-foot.* By C. WEIS.—*Copenhagen*, 1844. 12mo, pp. 94.

THIS is a small, unpretending dissertation in less than a hundred pages, containing more practical information than some of the more bulky tomes written on the same subject. The author gives the result of his experience, to which, under the several heads of *treatment*, *age for the operation*, *period of origin and causes of club-foot*, *complications*, *prognosis*, *preparatory treatment*, *modes of operating*, and *unlucky incidents during the treatment*, he has prefixed the opinions of Stromeyer, Dieffenbach, Scousetten, Duval, Guerin, Little—in short, of all the principal modern writers on treatment of deformities. We have so fully considered the applications of tenotomy, and the general treatment of deformities, in some former Numbers, that it is unnecessary to follow the Danish author through every division of the subject. We shall confine our remarks to some of the more essential questions discussed by him.

The author considers it is not advisable to delay the operation until the age of 12 months, as some authors have recommended. We agree with him to the extent that wherever the operation is absolutely requisite, the earlier it is performed the better; but we wish the author had given us precise rules for determining, during the earliest period of life, what cases may be successfully treated by mechanical means, and those which indispensably demand operation. We fear the mechanical orthopædists of the present day still profess to cure all by instruments, whilst the surgical orthopædists display a still greater zeal in performance of tenotomy in every case. We believe that if the cases of congenital club-foot be classed under three grades, all those of the first or slightest grade may unquestionably, within a reasonable time, be cured by well-applied mechanical treatment. Many of the second grade will also yield to this method, although the treatment will necessarily be more tedious, perhaps scarcely completed when the time arrives at which healthy children usually "walk alone,"—say at the age of 12 or 14 months. The majority of cases of the intermediate and the whole of those of the severer grades, can only be remedied within a reasonable time by operation. We should not venture our opinion in opposition to the evident leaning of the author more fully to appreciate the operative method, if we did not entertain the conviction, that, however essential tenotomy is in the above proportion of cases, it is improper in the remainder, for although the form of the member is by means of tenotomy more promptly remedied, the function of the divided muscles is often less completely restored. We have met with many instances of talipes successfully treated without tenotomy, in which the muscles of the calf attained the normal development, whereas, we believe that after tenotomy the muscles usually remain smaller than natural. We desire our views not to be misunderstood ; we are too sensible of the value of tenotomy, properly applied, to disparage it ; we simply offer an opinion based upon experience, that may possibly stay the hand of an over-zealous operator. Useless, i. e. improper or unnecessary tenotomy weakens the muscular powers of a limb which without operation might have attained normal development, whereas,—paradoxical as it may at first sight appear, —the proper application of section of tendons, by enabling the attendant to effect a cure of a deformity, proved by experience in similar cases to be otherwise irremediable, prevents the occurrence of atrophy, the ordinary concomitant of uncured club-foot ; nay more, in proper cases, the operator has the satisfaction of witnessing a gradual augmentation of muscle.

The following remarks occur under the head of "complications."

"Whenever talipes is complicated with hemiplegia, paraplegia, or contraction of other articulations, as for example, of the hip, knee, wrist, or hand, or when strabismus coexists, the assemblage of symptoms indicates a common origin in the brain, or more probably in the spinal cord. The treatment of the secondary affections cannot be contemplated, whilst the morbid process in the central organ is in actual progress, but upon removal of the primary disease, the treatment of the contracted limbs, or the strabismus, may be undertaken. Very commonly, however, it is exceedingly difficult to discriminate the cases in which the lesion in the nervous centre is still in progress, displaying its existence by the secondary affections, from those cases in which the contractions independently exist. It is necessary for the solution of this difficulty, to seek for the peculiar symptoms of disease of brain or spinal chord, to ascertain whether the secondary phenomena have long remained stationary, i. e. whether the contraction or paralysis of muscles already affected, continues to increase, or progressively involves others. Notwithstanding the utmost diligence, it may even be no easy matter to determine whether the aggravation depends upon the primary disease, or is the result of the existing deformity." (p. 33.)

We agree with Dr. Weis on the importance of determining these matters before undertaking the treatment of deformities, but we cannot consider that with due diligence and experience the matter is so difficult as he considers it to be. With the exception of some spasmodic contractions, which, like other convulsive diseases, such as tetanus, chorea, and cramps, are unaccompanied with evidence of physical lesion of the nervous tissue and febrile disturbance, deformities are for the most part well defined in their modes of origin. We can usually observe without difficulty the *incrementum* and *acme* of the disorder, as the older medical writers would have spoken. We can lay down a better rule for determining the period when the operation may with propriety be no longer delayed, namely, with very rare exceptions, never to divide tendons until satisfied that the resistance to proper movement of the articulations arises not simply from augmentation of contractility of the muscular fibres, but from structural or organic shortening of the fibres, (*retraction* of the French writers,) by which a mechanical obstacle to elongation is offered. We are convinced that tenotomy operates mechanically in the case of contractions, and not, as asserted by Stromeyer, dynamically; except in so far as the section must necessarily for a time, until close reunion of the divided parts takes place, dynamically weaken the muscular fibres, and when a wide separation of the divided parts permanently continues, the debilitating influence of the section must be permanent. Stromeyer has taught that tenotomy is antispasmodic in the therapeutic signification of the term.

Dr. Weis follows the original recommendation of Stromeyer, in causing his patients to wear the extension apparatus a few days before operation, by which means the patient becomes accustomed to the instrument, and the practitioner by observing its action can improve the adjustment without the inconvenience and loss of time occasioned by alterations after performance of the operation. This preliminary proceeding is not necessary to the practitioner experienced in treatment of deformities, but we are persuaded that if it had been followed by the inexperienced, fewer unsuccessful operations of tenotomy, and the direful sloughings from undue pressure, of which we have heard mention, and the consequent suffering and disappointment, would have been avoided. In the chapter on the tendons which require division, Dr. Weis strongly insists, in the treatment of T. varus, on the importance of dividing the muscles which act upon the front part of the foot and produce the powerful inversion which characterizes this affection, so as to overcome this part of the deformity before dividing the tendo achillis, and attempting to bind the foot. This plan, which originated with Dr. Little, has, no doubt, from its obvious division of the treatment of severe cases of varus into two stages greatly facilitating the result, suggested itself to every person familiar with the difficulties of these cases.

The author fully describes the modes of dividing the different tendons, a matter not difficult of execution, when those prominently or superficially situated are concerned.

We are glad to meet in the pages of our author the discussion of the methods of arriving at a perfect section of the posterior tibial tendon, the contraction of which so powerfully affects the form of the ankle. In members sparingly covered with adipose tissue, and in slight deformities, the posterior tibial tendon may with facility be attained, but in such cases the operation is seldom required. In infantile varus, on the contrary, especially when the affection reaches a high grade, or in the fat limb of a healthy infant, the section of this tendon is not a perfectly simple matter on account of the comparative depth at which it is situated, and its proximity to the posterior tibial blood-vessels and nerve.

Stromeyer effects the division by inserting the point of a knife between the artery and tendon one and a half inch above the internal malleolus, the artery being secured from injury by application of the index-finger upon it. He then directs the edge of a knife against the inner side of the tibia, and severs the tendon. Dr. Weis prefers the method of Velpeau, who divides the tendon close to its insertion into the navicular bone. The limb is rested on the outer side, a sharp pointed knife is introduced at the distance of a few lines from the malleolus, a little beneath and posterior to the *anterior* tibial tendon. The point is then carried directly backwards, and attains the tendon about an inch below and in front of the extremity of the malleolus. "Injury to the plantar artery is with certainty avoided by taking care that the point of the instrument is not carried further into the sole than absolutely necessary for section of the tendon." Now, it is precisely this dependence of the safety of the artery, upon not carrying the point of the knife too far into the sole, which, from our experience, leads us to decide that Velpeau's operation is less secure as regards the artery than Stromeyer's, and also less certain of effecting the object of the operation—complete division of the posterior tibial tendon. It should be an important *rule* in tenotomy to cut in a direction *away from* arteries and nerves, and not, as in Velpeau's operation, *towards* them. We should not omit to mention the result of Dr. Weis's experience: he states that he has found Velpeau's method safe in the performance and certain in the results. Our author is unacquainted with the plan of operation, known here as that of Dr. Little, which consists in introducing, about one inch above and in front of the tendon (through a puncture made in the integuments and fascia with a common scalpel,) a blunt-pointed knife similar to Bouvier's tenotome. This is passed beneath the tendon, towards which the *surface* of the instrument is directed, the operator then turns the *edge* towards the tendon whilst an assistant endeavours to straighten the member. The tendon then cuts itself, as it were, upon the opposing edge. This operation is safe, as in case of injury to the posterior tibial artery the vessel will be completely severed, and not punctured as when a pointed instrument is used. Experience in subcutaneous section of this tendon and artery has proved that in this mode of operation no hemorrhage ensues, whereas after puncture of the vessel much trouble and anxiety has been occasioned.

The treatise contains representations of various suitable apparatus, an omission we have noticed in other works, the authors of which evidently attach too great importance to the éclat of the surgical operation, whilst the absence of details respecting mechanical treatment indicates their inattention to this portion of treatment, admitted upon all hands to be as essential to recovery as the operation itself. Many failures may doubtless be traced to this source.

We take our leave of Dr. Weis in thanking him for the copious information his book contains, and in expressing our admiration of the candid, modest manner in which his opinions are advocated.

